# Incidence and Risk Factors for Hepatocellular Carcinoma in Texas Latinos: Implications for Prevention Research

**DOI:** 10.1371/journal.pone.0035573

**Published:** 2012-04-18

**Authors:** Amelie G. Ramirez, Nancy S. Weiss, Alan E. C. Holden, Lucina Suarez, Sharon P. Cooper, Edgar Munoz, Susan L. Naylor

**Affiliations:** 1 Institute for Health Promotion Research and Cancer Therapy and Research Center, University of Texas Health Science Center-San Antonio, San Antonio, Texas, United States of America; 2 Institute for Health Promotion Research, University of Texas Health Science Center-San Antonio, San Antonio, Texas, United States of America; 3 Department of Cellular and Structural Biology, University of Texas Health Science Center-San Antonio, San Antonio, Texas, United States of America; 4 Environmental Epidemiology and Disease Registries, Texas Department of State Health Services, Austin, Texas, United States of America; 5 School of Public Health-University of Texas Health Science Center-Houston, San Antonio, Texas, United States of America; Sookmyung Women's University, Republic of Korea

## Abstract

**Background:**

Hepatocellular carcinoma (HCC) is increasing in the U.S. despite a decline in cancer overall. Latinos have higher rates of HCC than the general population according to the Surveillance, Epidemiology, and End Results (SEER) Program. Not included in SEER, Texas Latinos make up one-fifth of the U.S. Latino population. To determine whether HCC incidence differs among U.S. and Texas Latinos, this descriptive study compares HCC incidence from 1995 through 2006 among three Latino populations: U.S. SEER, Texas overall and a South Texas subset. To identify lines of prevention research, we compare prevalence of known HCC risk factors among these Latino groups.

**Methods:**

Data were collected from the U.S. SEER Program, Texas Cancer Registry and Texas Department of State Health Services (TDSHS). Annual age-specific and age-adjusted HCC incidence rates, annual percent changes (APCs) and 95% confidence intervals were calculated as well as prevalence of obesity, diabetes, heavy alcohol use and cigarette smoking.

**Results:**

Of the three Latino groups compared, South Texas Latinos had the highest age-adjusted HCC incidence rates and SEER Latinos had the lowest (10.6/100,000 (10.1–11.1) and 7.5/100,000 (7.2–7.7), respectively). HCC incidence significantly increased over time (APCs>0) among Latinos in all three geographic groups. Between 1995 and 2006, there was an increase in obesity among all three populations, and obesity was highest among South Texas Latinos. Diabetes increased among U.S. Latinos, and Latino women in South Texas had significantly higher diabetes prevalence than U.S. Latino women. Cigarette smoking and heavy alcohol use were similar among groups.

**Conclusions:**

The incidence of HCC among Latinos in South Texas is higher than elsewhere in the United States. Higher rates of HCC among Texas and South Texas Latinos may be associated with greater prevalence of obesity and diabetes, risk factors for HCC that are amenable to intervention.

## Introduction

Hepatocellular carcinoma (HCC) is a global problem with increasing incidence in the U.S. for unknown reasons despite a decline in cancer overall during 1975–2006 [1], [2]. Because primary liver cancer is a growing concern, more attention should be given to addressing causes for this disease that are avoidable, preventable, or treatable. These include infection with the hepatitis B virus (HBV) or C virus (HCV), heavy alcohol consumption, diabetes, obesity, ingestion of aflatoxin or fumonisin, metabolic syndrome, nonalcoholic steatohepatitis and several rare exposures and metabolic disorders (hemochromatosis, α-1 antitrypsin deficiency, porphyrias) [3], [4], [5], [6], [7], [8].

Latinos have higher rates of HCC than the general population according to national data sources, including the Surveillance, Epidemiology, and End Results (SEER) program [9]. Texas is not included in the SEER registries, and yet Texas Latinos represent one-fifth of the total U.S. Latino population. This descriptive study compares HCC incidence and trends from 1995 to 2006 among Latinos from the U.S. SEER population with Latinos from two Texas populations—Texas overall and a South Texas subset that is nearly 70% Latino.

Texas Latinos are exposed to high rates of personal health behaviors and environmental risks for HCC [10]. With lower income and education levels, this exposure is especially pronounced in the more concentrated Latino population of South Texas [11]. With the goal of identifying lines of research that could be pursued to reduce HCC incidence, we examine the regional prevalence of four potential HCC risk factors (obesity, diabetes, alcohol consumption and cigarette smoking) among the Texas Latino population.

The specific purposes of this study are twofold. One is to determine whether HCC incidence for Latinos in Texas and South Texas differs from Latinos in the U.S. SEER population. Second is to compare the prevalence of probable HCC risk factors (obesity, diabetes, alcohol consumption and cigarette smoking) among Latinos in these three populations.

## Methods

This study is based on public use de-identified data from the U.S. SEER 13 Registries, Texas Cancer Registry [12] and Texas Department of State Health Services (TDSHS) [13]. The study did not require informed consent and was exempted from review by the University of Texas Health Science Center at San Antonio Institutional Review Board. The authors, however, obtained or submitted Limited-Use Data Agreements from SEER and Texas Cancer Registries (Weiss, Munoz) as well as to TDSHS (Holden).

### HCC incidence data

HCC incidence data were obtained from two sources: 1) U.S.SEER Registries and 2) Texas Cancer Registry. SEER is a population-based cancer registry system in certain areas of the U.S. including thirteen registries that have been part of SEER since 1995 or earlier. This study used the SEER 13 grouping, which includes registries from Connecticut, Hawaii, Iowa, New Mexico, Utah, metropolitan Atlanta, Detroit, Los Angeles, San Francisco-Oakland, San Jose-Monterey, Seattle-Puget Sound, rural Georgia and Alaska. The Texas Cancer Registry is an identically-organized, population-based registry of all 254 Texas counties and follows all standards and coding criteria of the SEER dataset including possession of the North American Association of Central Cancer Registries (NAACCR) Gold Certification.

Population denominators used for all rate calculations were those available from the NCI SEER program, adjusted for Hurricane Katrina but not for delay in case reporting [12]. HCC incident cases from 1995 through 2006 were selected for Latino and non-Latino White (NLW) male and female residents of the 13 SEER registries (cumulative population at risk = 93,078,598 Latino; 374,653,050 NLW); Texas Cancer Registry (cumulative population at risk = 82,256,301 Latino; 171,621,171 NLW for all of Texas) and Texas Cancer Registry (cumulative population at risk = 29,000,316 Latino; 15,684,300 NLW for the 38 counties comprising South Texas).

### Classification of malignancies

HCCs were identified by site code C22, tumor behavior code “malignant" and ICD-O-2 histology codes 8170–8175. All morphology codes other than these were excluded. As of January 1, 2001, all cases reported to SEER were required to have an ICD-O-3 histology and behavior code. Because cases diagnosed prior to this date used the ICD-O-2 coding scheme, analysis required that all data be placed on the same coding system. We used ICD-O-2 codes provided by SEER after the application of the ICD Conversion Program to convert relevant source records from ICD-O-3 format to ICD-O-2 format [14].

### Demographic data

For all groups compared, ethnicity was defined using the NAACCR Hispanic/Latino Identification Algorithm, version 2 [15], and urban/rural residence was identified using the U.S. Department of Agriculture 2003 Urban/Rural Continuum criteria [16]. Metropolitan counties with continuum codes 1–3 were designated urban and non-metropolitan counties with codes 4–9 rural.

### Risk-factor data

US-level and Texas aggregate county-level behavioral risk factor data were obtained from the TDSHS [13]. These data were subsets created by the TDSHS from the CDC's Behavioral Risk Factor Surveillance System. Behavioral risk factors included obesity (Body Mass Index (BMI)≥30 kg/m^2^), heavy alcohol use (>2 drinks/day for men, >1 drink/day for women), cigarette smoking (current smoker) and diagnosed non-gestational diabetes [13].

### Statistical analysis

SEER*Stat software v 6.5.1 (SEER*Stat, National Institutes of Health) generated 1995–2006 average annual age-specific and age-adjusted HCC incidence rates, rate ratios (RR), annual percent changes (APCs) and 95% confidence intervals (CI) for Latino and NLW populations in the SEER, Texas and South Texas datasets. APCs were derived using weighted least squares point-estimation; trends were tested for statistical significance using SEER*Stat. Risk factor prevalence estimates and CI were calculated utilizing SPSS Complex Samples software v 17.0 (SPSS Inc., Chicago Ill). Differences were assessed at p<.05 if confidence levels did not overlap.

## Results

### Case characteristics

From 1995 through 2006, there were more HCC cases in Texas Latinos than SEER Latinos (Table 1). There were 3,374 Latino HCC cases diagnosed in SEER, 3,891 in Texas, and 2,011 in South Texas. Latinos accounted for more than a third of HCC in Texas and nearly three-fourths of all HCC in South Texas, higher proportions than in SEER (17%). These higher proportions of Latino HCC cases in Texas and South Texas are commensurate with higher proportions of Latinos in the general population of these areas. More than 70% of HCC in Latinos occurred in men, with similar percentages observed among SEER, Texas and South Texas groups. South Texas Latinos were diagnosed with HCC at older ages than SEER Latinos. The median ages at diagnosis were 62, 65 and 67 years for SEER, Texas and South Texas respectively. A larger proportion of HCC occurred among rural South Texas (14.8%) and Texas Latinos (14.3%) compared to SEER Latinos (5.0%).

**Table 1:** Case Characteristics of HCC in Latinos from US SEER, Texas and South Texas, 1995–2006.

	US SEER		Texas		South Texas	
	N	%Total	N	%Total	N	%Total
HCC Cases	19,966		10,341		2,772	
Population at Risk	467,731,648		253,877,472		44,684,616	
Latino Cases	3,374	16.9	3,891	37.6	2,011	72.5
Latino Population at Risk	93,078,598	19.9	82,256,301	32.4	29,000,316	64.9
Age at Diagnosis (years)						
<40	85	2.5	79	2.0	28	1.4
40–49	438	13.0	477	12.3	182	9.1
50–59	931	27.6	874	22.5	384	19.1
60–69	867	25.7	1,003	25.8	561	27.9
70–79	778	23.1	978	25.1	562	27.9
80–84	156	4.6	271	7.0	163	8.1
85+	119	3.5	209	5.4	131	6.5
Age/Diagnosis (years)	median = 62	median = 65	median = 67			
Sex						
Male	2,487	73.7	2,801	72.0	1,445	71.9
Female	887	26.3	1,090	28.0	566	28.1
Residence						
Urban	3,206	95.0	3,333	85.7	1,714	85.2
Rural	167	5.0	558	14.3	297	14.8

### Incidence

South Texas Latinos had the highest overall HCC incidence rates regardless of age or gender (Table 2). The HCC incidence rate of Latinos in South Texas was 10.6/100,000 (10.1–11.1) and the rate among SEER Latinos was 7.5/100,000 (7.2–7.7). HCC incidence was highest in South Texas Latino men and women (17.3/100,000 and 5.4/100,000), more than 45% and 42% higher than in respective SEER subjects. Latinos in Texas and South Texas had a significantly greater relative risk of HCC than SEER Latinos, and the risk was greatest in South Texas (Table 2). Compared to the SEER population, the rate ratios of age-adjusted HCC incidence rates for Latinos were 1.27 (1.21–1.33) in Texas and 1.42 (1.34–1.51) in South Texas, respectively.

**Table 2:** Incidence Rates^1^ and Rate Ratios (RR) of HCC in Latinos from US SEER, Texas and South Texas, 1995–2006.

	US SEER			Texas			South Texas			
	Gender	N	Rate^1^ (95% CI)	RR	N	Rate^1^ (95% CI)	RR (95% CI)	N	Rate^1^ (95% CI)	RR (95% CI)
Latinos	Male	2,487	11.9 (11.4–12.4)	1.00	2,801	14.8 (14.2–15.4)	1.24 (1.17–1.32)	1,445	17.3 (16.4–18.2)	1.45 (1.4–1.6)
	Female	887	3.8 (3.6–4.1)	1.00	1.090	5.1 (4.8–5.4)	1.34 (1.2–1.5)	566	5.4 (5.0–5.9)	1.42 (1.27–1.58)
	Total	3,374	7.5 (7.2–7.7)	1.00	3,891	9.5 (9.2–9.8)	1.27 (1.21–1.33)	2,011	10.6 (10.1–11.1)	1.42 (1.34–1.51)
NLW^2^	Male	6,516	4.8 (4.7–4.9)	1.00	3,470	5.2 (5.1–5.4)	1.09 (1.05–1.14)	479	6.0 (5.4–6.5)	1.24 (1.13–1.37)
	Female	2,181	1.3 (1.3–1.4)	1.00	1,123	1.4 (1.3–1.5)	1.07 (.99–1.15)	169	1.7 (1.4–2.0)	1.30 (1.10–1.53)
	Total	8,697	2.9 (2.8–3.0)	1.00	4,593	3.1 (3.1–3.2)	1.08 (1.04–1.12)	648	3.7 (3.4–4.0)	1.27 (1.17–1.37)

1 Rates per 100,000 and age-adjusted to the 2000 US Standard Population (19 age groups).

2 Data for non-Latino whites (NLW) is included for general comparison.

**Figure 1:**
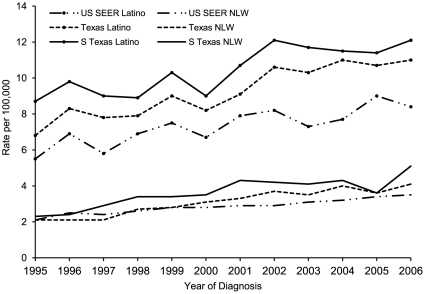
Annual Age-adjusted incidence rates of hepatocellular carcinoma by ethnicity, 1995–2006. Annual age-adjusted incidence of HCC increased over the study period and was highest among South Texas Latinos. Data for non-Latino whites (NLW) is included for general comparison purposes.

**Figure 2:**
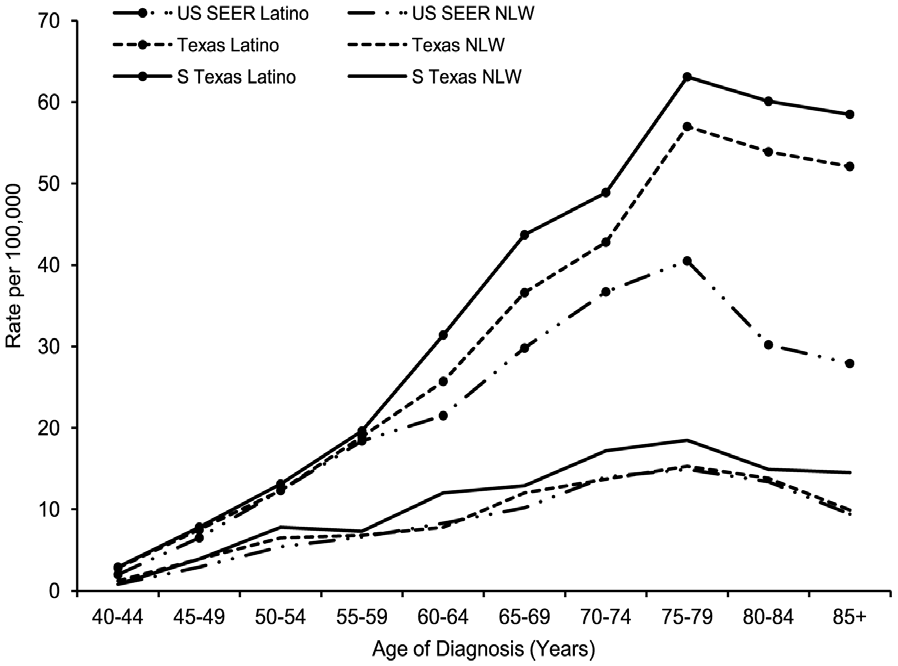
Age-specific incidence rates of hepatocellular carcinoma. Age-specific incidence increases with age from 40 to 79 years. Data for non-Latino whites (NLW) is included for general comparison purposes.

From 1995 to 2006, annual age-adjusted HCC incidence rates were consistently higher among South Texas and Texas Latinos than SEER Latinos (Figure 1). Over the study period, age-specific HCC incidence among all groups became greater with increasing age from 40 to 79 years (Figure 2), and age-specific rates peaked at 75–79 years. South Texas Latinos had the highest age-specific rates, significantly higher than SEER Latinos for those aged 60 and older.

### Trends

HCC incidence significantly increased over time (APCs>0) among Latinos in all three geographic groups (Table 3). Of interest, all age groups from 50–59 years experienced higher percent changes in HCC incidence than older age groups. There were no significant differences in trends among Latino groups in the three areas.

**Table 3:** Annual percent change (APC) of HCC incidence^1^ from 1995 to 2006 by age for US SEER, Texas and South Texas.

		US SEER	Texas	South Texas
	AGE (years)	APC (%) (95% CI)	APC (%) (95% CI)	APC (%) (95% CI)
Latino	All ages	3.3* (1.8–4.8)	4.1* (2.9–5.2)	3.0* (1.7–4.3)
	50–59	7.1* (4.8–9.4)	9.2* (6.7–11.8)	7.9* (4.8–11.1)
	60–69	1.3 (−1.0–3.7)	3.5* (0.2–6.8)	2.6 (−1.2–6.4)
	70–79	3.6* (1.4–5.9)	3.3* (1.3–5.2)	2.6* (0.8–4.5)
	80–84	3.3 (−3.2–10.2)	0.1 (−4.0–4.4)	0.7 (−2.9–4.4)
	85+	−4.6 (−8.9–0.1)	2.3 (−2.3–7.3)	∧
NLW^2^	All ages	3.9* (3.2–4.6)	6.6* (5.0–8.2)	5.9* (3.5–8.3)
	50–59	12.1* (10.5–13.7)	14.5* (11.6–17.4)	∧
	60–69	2.0* (0.5–3.4)	3.2* (1.1–5.4)	4.3 (−0.2–9.0)
	70–79	1.4* (0.4–2.5)	4.3* (2.0–6.7)	3.0 (−0.8–6.9)
	80–84	2.3 (−0.3–5.1)	2.3 (−0.5–5.1)	∧
	85+	1.3 (−0.6–3.2)	5.6* (0.8–10.6)	∧

1 Incidence rates are age-adjusted for all ages and unadjusted for specific age groups.

2 Data for non-Latino whites (NLW) is included for general comparison.

* Significantly increasing trend (p<.05).

APC = Annual Percent Change.

CI = Confidence Interval.

∧ Less than 6 cases for one or more years.

### Risk factors

**Figure 3:**
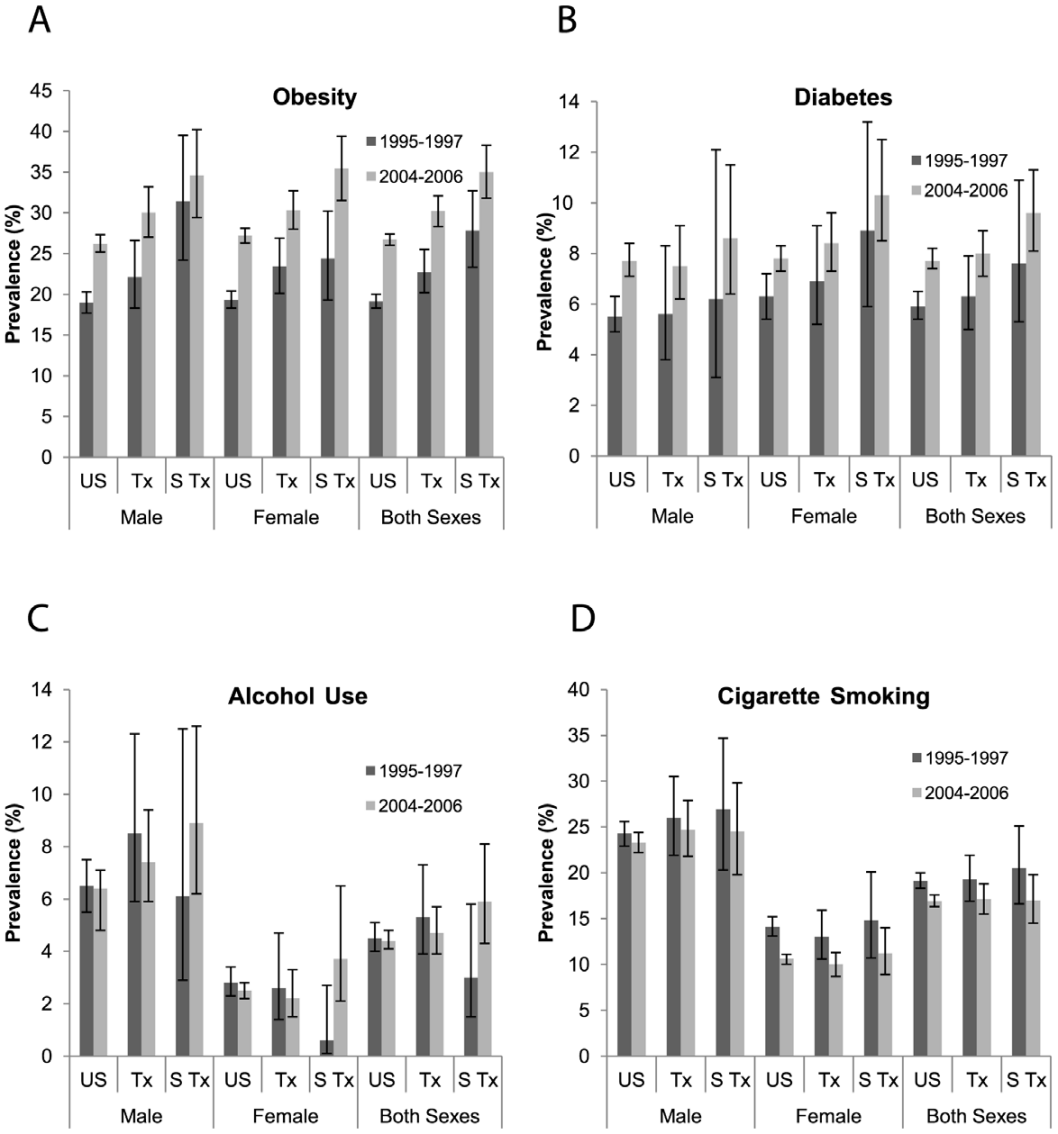
HCC risk factor prevalence among U.S., Texas and South Texas Latinos, 1995–1997 and 2004–2006. A) Obesity prevalence increased among all three populations of Latinos and was highest among Latinos in South Texas. B) Diabetes prevalence significantly increased among U.S. Latinos. There were also non-significant increases among Texas and South Texas Latinos. Latino women in South Texas had significantly higher diabetes prevalence than U.S. Latino women. C) Heavy alcohol use did not change significantly over time among any Latino group, though there were non-significant increases in South Texas. D) Cigarette smoking significantly declined only among U.S. Latinos. During 2004–2006, there were no significant differences among the three Latino groups.

Prevalence percentages of HCC-related behavioral risk factors for Latinos in the U.S., Texas and South Texas for two time periods, 1995–1997 and 2004–2006 are compared in the four panels of Figure 3. Obesity increased among all three groups of Latinos. Texas and South Texas Latinos had higher obesity prevalence than U.S. Latinos during the most recent period (30.2% and 35.0% versus 26.7%). Additionally, diabetes prevalence increased among U.S. Latinos. Texas and South Texas Latinos also showed an increasing pattern, although confidence intervals overlapped. For 2004–2006, the prevalence of diabetes was higher in South Texas Latino women than U.S. Latino women (10.3% and 7.8%, respectively). Heavy alcohol use did not change significantly over time among any Latino group and only U.S. Latinos had a decline in the prevalence of cigarette smoking. During 2004–2006, cigarette smoking and heavy alcohol use were similar among the three Latino groups.

## Discussion

Results from this study support the observations of others that HCC is on the rise in the United States [2], [9], [17], [18]. Our primary finding is that, among the three Latino populations compared, the incidence of HCC was highest among Latinos in South Texas. Between 1995 and 2006, age-specific HCC incidence rates increased from 40 to 79 years, and these increases were greater among Texas and South Texas Latinos than among those from SEER areas. The greatest APC in the incidence of HCC between 1995 and 2006 occurred among Latinos between 50 to 59 years of age.

The comparison of probable HCC risk factors shows that there was an increase in obesity among all three populations of Latinos and that obesity was highest among South Texas Latinos. We also found an increase in diabetes prevalence among U.S. Latinos and non-significant increases in diabetes prevalence among Texas and South Texas Latinos. Further, Latino women in South Texas had significantly higher diabetes prevalence than U.S. Latino women. We found no significant changes in heavy alcohol use among any Latino group, and only U.S. Latinos showed decline in the prevalence of cigarette smoking. Cigarette smoking and heavy alcohol use were similar among the three Latino groups.

Increasing diabetes and obesity prevalence may be relevant to the development of HCC in South Texas Latinos. The prevalence of these risk factors is higher in South Texas Latinos, so the attributable risk of HCC due to diabetes and obesity may be greater. The CDC reports that Latino adults 18 years of age and older are 1.2 times more likely to be obese than NLW, and among children aged 6–17 years of age Latinos are 1.4 times more likely to be obese than NLW [19], indicating an upward trend that may impact differential rates of obesity-related diseases including HCC. A recent study reported a 2-fold increased HCC risk in obese subjects, a 4-fold increased risk in diabetics and a 5-fold increased risk for obese diabetics, after adjustment for other known risk factors including infection with hepatitis B and C viruses [20]. Although the study's sample size was modest, 37% of HCC cases without HBV and/or HCV infections were attributed to diabetes and obesity combined.

We speculate that Texas Latinos experience more obesity, diabetes and HCC than other Latinos because of cultural history, socioeconomic factors and maybe genetic predisposition. The composition of the Latino subgroups in Texas and SEER regions may differ, and their cultural traditions and immigration status may modify or impact the risk of cancer [21]. In the U.S. overall, about 64% of Latinos are of Mexican origin; however, in Texas and South Texas, nearly 85% of Latinos are of Mexican origin [22], [23]. During the 20^th^ century, regional policies promising better housing, food and jobs attracted Mexican immigrants to South Texas where they subsequently adopted a more sedentary lifestyle and an Americanized diet that consisted of more fat and simple carbohydrates, less complex carbohydrates and less nutrient-dense vegetables and fruits [24]. Additionally, about a third of the Mexican American gene pool is derived from Native American sources [25], [26] and since the latter may have a genetic predisposition to attributable risks for HCC [27], Mexican Americans could share this, and with it the likelihood of elevated rates of diabetes, obesity and alcohol use [21]. We have evidence for example, that the so-called “reward" genotypes D2 dopamine receptor Taq 1A genotypes (A1A1, A1A2) have been associated with obesity, diabetes, alcohol and tobacco use as well as a variety of other problems [28].

A limitation of this study is that we do not have incidence data for hepatitis infection. HCC has long been associated with the hepatitis B and C viruses, focusing HCC etiology research on them and forecasting upward trends in HCC [29], [30], [31]. A study of the prevalence of chronic HCV infection in Texas from 1988–1994 reported that 1.8% of all Texans were infected with HCV, 1.4% among whites and 2.0% among Latinos [32]. Hepatitis infection is likely a strong risk factor for development of a proportion of Latino HCC cases; however, these attributable risks cannot alone explain the rising trends in HCC nor the differences in HCC incidence among the Latino groups compared in our study. Thus, productive avenues of HCC research should target not only hepatitis prevention, but also other preventable risk factors such as diabetes, obesity and heavy alcohol consumption.

To our knowledge, this is the first study to show increasing incidence of HCC in South Texas as well as increasing HCC risk factors among Latinos in this area. Given that 20% of U.S. Latinos reside in Texas, two-thirds of the population of South Texas is Latino (mainly of Mexican origin), and half of the HCC incident cases in Texas occur in South Texas, we have prioritized risks for HCC that may result in higher rates of the disease in this group, particularly diabetes and obesity. Although Latinos in Texas are no more likely than others to engage in excessive alcohol use or cigarette smoking, these behaviors remain as important risk factors for HCC. However, prevention efforts for Latinos in Texas should concentrate on addressing diabetes and obesity.

Most importantly, the risks we have identified are amenable to intervention. Clearly there is a need to focus on HCC prevention research and intervention which takes into account not only risks for the disease, but also genetic, cultural and socioeconomic predisposing features that may mediate the exposure-disease relationship. By understanding the components of cultural adaptation which influence health and disease, modifiable factors can be identified, populations at high risk can be targeted, and interventions can be tailored to fit the specific components affecting risk. Future studies using multifocal research and subsequent culturally sensitive intervention design, should target diabetes, obesity and other known factors related to liver cancer.

In summary, this study documents that the incidence of HCC among Latinos in South Texas is significantly higher than elsewhere in the United States. Higher rates of HCC among Texas and South Texas Latinos are likely the result of increased risks such as obesity and diabetes. Each of these has been shown to be a significant attributable risk for HCC among Latinos, and may be a consequence of cultural characteristics of this population. This indicates a need for further research to inform tailored prevention efforts directed at these risks among Latinos.
